# The First Result of Relative Positioning and Velocity Estimation Based on CAPS

**DOI:** 10.3390/s18051528

**Published:** 2018-05-12

**Authors:** Jiaojiao Zhao, Zishen Li, Jian Ge, Liang Wang, Ningbo Wang, Kai Zhou, Hong Yuan

**Affiliations:** 1 Academy of Opto-Electronics, Chinese Academy of Sciences, No.9 Dengzhuang South Road, Haidian District, 100094 Beijing, China; zhaojj@aoe.ac.cn (J.Z.); jian.ge@aoe.ac.cn (J.G.); wangliang115@mails.ucas.ac.cn (L.W.); wangningbo@aoe.ac.cn (N.W.); zhoukai@aoe.ac.cn (K.Z.); yuanh@aoe.ac.cn (H.Y.); 2 School of Electronic, Electrical and Communication Engineering, University of Chinese Academy of Sciences, No.19A Yuquan Road, Shijingshan District, 100049 Beijing, China; 3 State Key Laboratory of Geodesy and Earth’s Dynamics, Institute of Geodesy and Geophysics, Chinese Academy of Sciences, No.340 Xudong Road, Wuchang District, 430077 Wuhan, China

**Keywords:** Chinese Area Positioning System (CAPS), relative positioning, velocity estimation

## Abstract

The Chinese Area Positioning System (CAPS) is a new positioning system developed by the Chinese Academy of Sciences based on the communication satellites in geosynchronous orbit. The CAPS has been regarded as a pilot system to test the new technology for the design, construction and update of the BeiDou Navigation Satellite System (BDS). The system structure of CAPS, including the space, ground control station and user segments, is almost like the traditional Global Navigation Satellite Systems (GNSSs), but with the clock on the ground, the navigation signal in C waveband, and different principles of operation. The major difference is that the CAPS navigation signal is first generated at the ground control station, before being transmitted to the satellite in orbit and finally forwarded by the communication satellite transponder to the user. This design moves the clock from the satellite in orbit to the ground. The clock error can therefore be easily controlled and mitigated to improve the positioning accuracy. This paper will present the performance of CAPS-based relative positioning and velocity estimation as assessed in Beijing, China. The numerical results show that, (1) the accuracies of relative positioning, using only code measurements, are 1.25 and 1.8 m in the horizontal and vertical components, respectively; (2) meanwhile, they are about 2.83 and 3.15 cm in static mode and 6.31 and 10.78 cm in kinematic mode, respectively, when using the carrier-phase measurements with ambiguities fixed; and (3) the accuracy of the velocity estimation is about 0.04 and 0.11 m/s in static and kinematic modes, respectively. These results indicate the potential application of CAPS for high-precision positioning and velocity estimation and the availability of a new navigation mode based on communication satellites.

## 1. Introduction

With the highly accurate performance of Positioning, Navigation and Timing (PNT), Global Navigation Satellite Systems (GNSSs), being of all-weather and real-time operational PNT service over land, sea, and even aerospace, have been developed rapidly around the globe [1]. Currently, the main GNSSs include the American GPS (Global Positioning System), the Russian GLONASS (Global Navigation Satellite System), the European Galileo and the Chinese BDS (Beidou Satellite Navigation System) [2]. These systems are featured by specialized satellites that can broadcast signals with navigation messages to users to estimate PNT parameters, such as location, velocity and time. However, there are also some challenges that have to be faced by the growing development of GNSS systems [3], i.e., (1) service capabilities through positioning, velocity estimation and time service without communication, (2) the weakness of the received signal, with fixed satellites/frequencies/code making the signal vulnerable to interference, and (3) the PNT accuracy of current GNSSs is significantly dependent on the performance of satellite clock which is extremely difficult to be kept stable in orbit [4]. Thus, it has for a long time been a global dream to design and establish a satellite-based system which can not only integrate the functions of communication and navigation, but also avoid the aforementioned problems. In view of this, Ai et al. [3,5] from the National Astronomical Observatories of the Chinese Academy of Sciences proposed an innovational navigation concept based on communication satellites including the GEO (geostationary orbit), DGEO (decommissioned geostationary orbit) and IGSO (inclined geosynchronous orbit) satellites in 2002. This proposed concept has been named the Chinese Area Positioning System (CAPS).

In the CAPS, the ranging code, time and navigation messages are generated and uplinked directly to the satellite from the ground control station, before being downlinked and broadcasted to users via the communication satellite transponder [5]. Compared with the traditional GNSSs like GPS, BDS, GLONASS and Galileo, CAPS does not require the installation of an atomic clock on satellites in orbit and could use a higher stability atomic clock in the ground station, since the navigation signal is generated in the ground control station. Moreover, CAPS can be flexibly established by renting the transponders on the communication satellites [6]. As a result, it significantly reduces costs and saves the system construction time when compared with the traditional GNSSs. In addition, CAPS uses C-band frequencies to transmit the navigation signals and is designed as a dual-frequency system. The frequencies of downlink signals are designed on 3862.02 and 4143.15 MHz as C1 and C2 frequencies [7,8]. Currently, only the C1 frequency is available. The special security code with a rate of 10.23 MHz is modulated on the C1 frequency. The chip length of the CAPS signal is about 30 m. Since the wavelength of the CAPS signal is about 1/3 of the GPS L2 signal (the wavelength of the CAPS C1 signal is 7.8 cm while that of the GPS L2 signal is about 24.4 cm), the precision of the carrier-phase measurement would be theoretically higher than that of the L-band. This feature is very advantageous for achieving positioning, velocity estimation and timing with a relatively high accuracy.

CAPS was first demonstrated by the Chinese Academy of Sciences based on two rented communication satellites from China Satellite Communications Co., Ltd. in 2005. Based on this demonstration, some experiments had been carried out from 2006 to 2010 by integrating the communication and navigation function in vehicles and ships from different areas over land and sea. Around 2011, CAPS was selected as the essential part of the BDS experiment system and aimed to support the BDS construction and development by testing the new or on-going navigation technology. CAPS is also an independent system with a PNT service capability, and it is currently in a development and testing phase [9]. The navigation signal on the C1 frequency of CAPS can be tracked by our self-developed receiver, and the code, carrier-phase and doppler measurements can be produced. The performance of Standard Point Positioning (SPP) based on CAPS was assessed by the simulated and real data in the previous studies [5,10] and the results showed that CAPS can independently achieve the SPP with an accuracy of 10 to 20 m, which was comparable to the SPP performance of traditional GNSSs.

To further validate the potential performance of CAPS in high-precision positioning and velocity estimation, this contribution is the first that assesses the relative positioning and velocity estimation performance of CAPS based on real data. A brief introduction to the system’s architecture, the observation equations and the relative positioning model of CAPS are described in Section 2. In Section 3, the noises of code and carrier-phase measurements are analyzed with the data from a zero-baseline experiment, and the accuracies of the relative positioning and velocity estimation are estimated, respectively, in static and kinematic modes. The conclusion and future work are drawn out in the final section.

## 2. CAPS Structure and Mathematical Model of Observations

CAPS is a new concept of satellite-based positioning system and it is quite different than the concept of traditional GNSSs. This section will give a very brief introduction of CAPS, including the operating principle and the original observation model for positioning.

### 2.1. System Structure of CAPS

**Figure 1:**
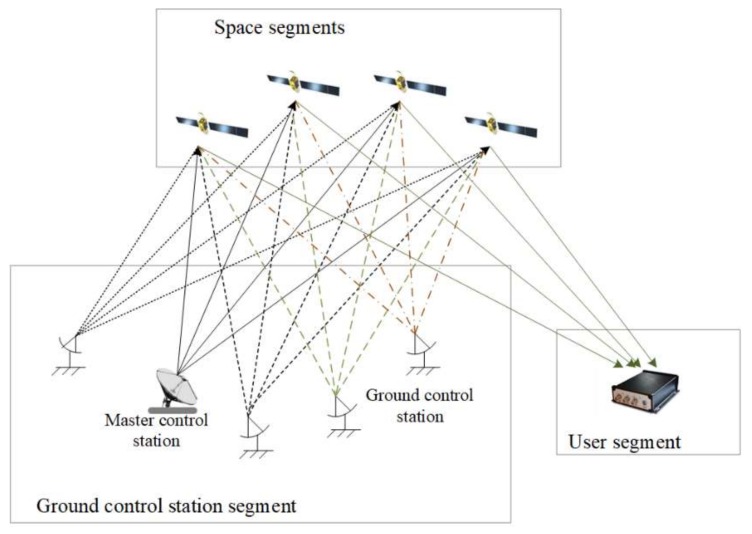
Basic system structure of the current CAPS test system in development.

**Figure 2:**
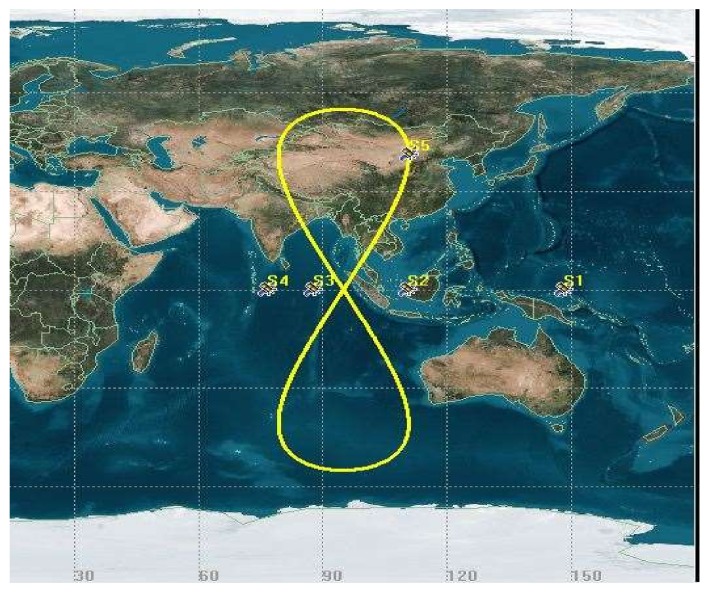
Ground track of CAPS satellites in orbit.

The basic structure of CAPS, consisting of the space, ground control station and user segments, is shown in Figure 1 [10]. The space segment includes GEO and IGSO communication satellites that receive and forward the navigation signal generated at the ground control station. There were four GEO satellites and one IGSO satellite available at the time of our experiment (see Figure 2). The ground control station segment is mainly responsible for terrestrial satellite monitoring and control, high-precision satellite orbit determination, navigation signal generation and transmission, time-frequency reference and communication. One of the ground control station is selected as the master control station. The user segment indicates all kinds of rover receivers, and it can track the navigation signal, communicate with the satellite, produce the raw measurement and achieve the positioning and timing.

It should be noted that CAPS has its own time system and coordinate frame. The time system is called the CAPS Time System (CAPST). CAPST traces UTC to the source, which is maintained by the National Time Service Center (NTSC). CAPST and UTC (NTSC) maintain a very good level of synchronization, with the bias between them remaining within 10 ns. The used coordinate frame is CAPS03, which is based on ITRF2000 [3].

It is well-known that the clock is the core part of a navigation system based on range measuring. In traditional GNSSs, the atomic clocks are generally placed in the satellites. On the contrary, the atomic clock in CAPS is placed in the master control station. Thus, the space and time reference in CAPS will be separated in the satellite and the ground. The orbit of the communication satellite in CAPS can be precisely determined using the data from ground tracking stations, and the clock offset resulting from the atomic clock can be easily controlled, and can be mitigated as much as possible during the navigation signal generation on ground. The navigation signal is generated with the ephemeris message based on the ground atomic clock in the master control station and sent to different communication satellites in CAPS. This navigation signal will be directly forwarded to the user using the existing transponder once the satellites receive the navigation signals from the master control station. To handle the distance and ground clock offset, a virtual clock technique is adopted in the ephemeris design of CAPS [11,12]. According to this situation, the maintenance of atomic clock becomes very easy when compared to the installation of an atomic clock on a satellite, and the atomic clock in the master control station can even be changed at any time to a new one if some new navigation technology needs to be tested.

### 2.2. Raw Observation Model

**Figure 3:**
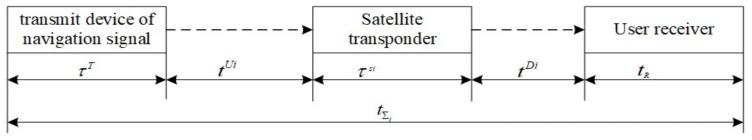
Total propagation time of the CAPS navigation signal from the ground control station to the user.

Based on the CAPS navigation signal from each individual CAPS satellite tracked by the receiver, the user can obtain the propagation time from the master control station to the user through each satellite. The satellite orbit, virtual clock model, and atmospheric model can be decoded from the received ephemeris message. The total propagation time of the navigation signal from the master control station to the satellite and then to the user can be divided into five parts shown by Figure 3 and Equation (1) with a time unit.

(1) $$t_{\Sigma_{i}}=\tau^{T}+t^{Ui}+\tau^{{}_{si}}+t^{Di}+t_{{}^{R}}+\varepsilon_{i}$$

where $t_{\Sigma_{i}}$ is the total propagation time of the CAPS navigation signal from the ground control station to the satellite and then to the user, and can be transformed to a code measurement by multiplying it by the speed of light; i is the index of the satellite tracked by the user receiver; $\tau^{T}$ is the hardware delay of the transmit device at the ground control station; $t^{Ui}$ is the propagation time from the transmit antenna at the master control station to the satellite antenna, including the ionospheric and tropospheric delays; $\tau^{{}_{si}}$ is the time delay of the satellite transponder, which differs from satellite to satellite; $t^{Di}$ is the propagation time from the satellite transponder antenna to the user antenna, including the ionospheric and tropospheric delays; $t_{{}^{R}}$ is the hardware delay and the clock offset of the user receiver; and $\varepsilon_{i}$ is the sum of the measurement noise and other unmodeled errors. $\tau^{T}$ and $\tau^{{}_{si}}$ can be generally calibrated in the laboratory and $t^{Ui}$ can be calculated once the coordinates of ground the control station and the satellite are precisely known and the atmospheric delay in the uplink path could be estimated precisely. Equation (1) can therefore be rewritten as:

(2) $$\left\{ \begin{array}{l} t^{Di}=t_{\Sigma_{i}}-T-t_{{}^{R}}-\varepsilon_{i}\\ T\;=\;\tau^{T}+t^{Ui}+\tau^{{}_{si}} \end{array}\right.$$

In CAPS, the virtual clock approach proposed by Wu et al. [11,12] is used and a polynomial function of 4th order is introduced to characterize the value and its variation of T in the time domain. The coefficients of this polynomial function with a 30-s update interval are broadcast to users by the ephemeris message. The CAPS receiver measures the $t_{\Sigma_{i}}$ with a tracked navigation signal, calculates the value of T with the model coefficients and removes it from $t_{\Sigma_{i}}$. In this way, the receiver can measure the propagation time from the satellite transponder antenna to the receiver antenna and translate it into code pseudo-range. Therefore, the navigation signal can be considered as being sent directly from the satellite, there is a virtual satellite clock on the satellite, and T can be considered as the offset of that virtual satellite clock.

**Figure 4:**
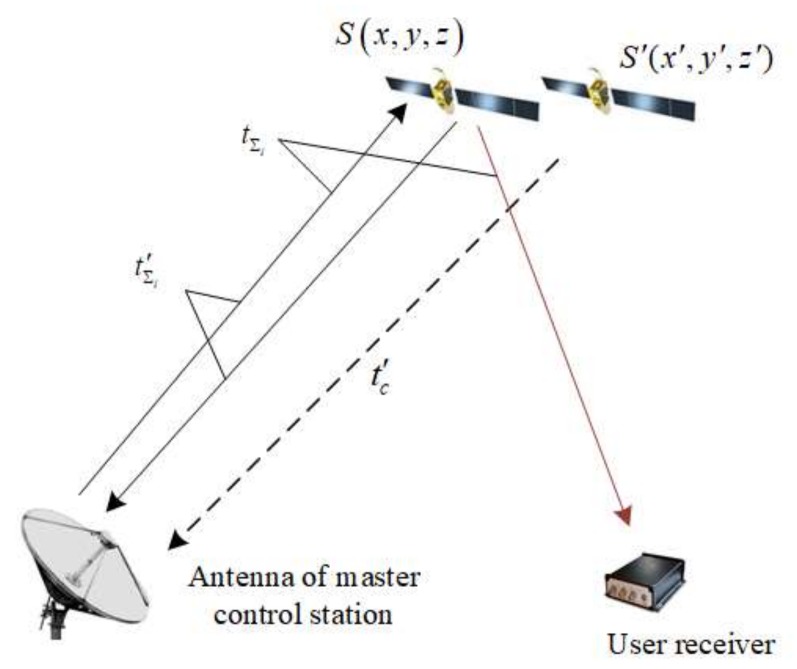
The calculation of the virtual satellite clock (S(x,y,z) is the true position of the satellite, and $S^{\prime }(x^{\prime },y^{\prime },z^{\prime })$ is the calculated position of the satellite with the ephemeris message).

To remove the effect of ionospheric and tropospheric delays in the uploading path, T was, in practice, calculated as a whole. The calculation of T is shown in Figure 4. The antenna with a large aperture uploading the navigation signal at the master control station can simultaneously receive the downlink signal of the C-band frequency from the satellite transponder. The master control station measures the total propagation time $t_{\Sigma_{i}}^{\prime }$. Obviously, $t_{\Sigma_{i}}^{\prime }$ contains the ionospheric delay in the uplink and downlink paths, the tropospheric delay in the uplink and downlink paths, the time delay of the satellite transponder, the hardware delay of the transmit and receive devices at the master control station. The distance from the satellite antenna to the antenna of the master control station can be calculated by the coordinates of the master control station which are precisely known and the coordinates of the satellite which are calculated from the ephemeris message. The distance was transformed to $t_{c}^{\prime }$ by dividing it by the speed of light. It should be noted that the coordinates of the satellite generally contain some bias from the ephemeris estimation error. Following this, *T* was calculated via:

(3) $$T=t_{\Sigma_{i}}^{\prime }-(t_{c}^{\prime }+t_{signac})$$

where $t_{signac}$ is the Sagnac effect in the downlink path which could be precisely calculated with the coordinates of satellite and the master control station. This means that for a receiver at the master control station, if T is removed from the measured $t_{\Sigma_{i}}^{\prime }$, the ionospheric delay in the uplink and downlink, the tropospheric delay in uplink and downlink, the time delay of the satellite transponder, and the hardware delay of the transmit and receive devices were all removed. The remaining error contains the Sagnac and ephemeris errors. Regarding the user receiver far from the master control station, the remaining errors mainly consists of the Sagnac error, the ephemeris error, the multipath errors, and the residual errors of ionosphere and troposphere after removing T from the measured $t_{\Sigma_{i}}$. It should be mentioned that the residual error of ionosphere and troposphere is resulted from the differences of the propagation paths of the downlink signal from the CAPS satellite respectively to the master control station and the user receiver. Generally, the residual errors of ionosphere and troposphere may become larger with the increase of the distance between the master control station and user receiver. To make it easier to understand this, we still refer to them ionospheric and tropospheric error later in this article.

In this way, the code pseudo-range measurement $\rho^{i}$ can be obtained by removing T from the measured $t_{\Sigma_{i}}$. Taking the ephemeris error, the bias resulting from the virtual clock offset model, the multipath error, and the ionospheric and tropospheric errors into account, the code pseudo-range can be described as:

(4) $$\rho^{i}=r^{i}+ct_{u}-ct^{i}-\tau_{iono}-\tau_{trop}-\tau_{mul,\rho }+\varepsilon_{i,\rho }$$

where $r^{{}_{i}}$ is the geometric distance between the CAPS satellite and the user receiver, c is the speed of light; and $t^{{}_{i}}$ is the sum of the model error of the virtual clock offset and the ephemeris error. The model error of the virtual clock offset represents the estimation error of T with the polynomial model; $t_{u}$ is the sum of the hardware delay and the clock error at the user receiver; $\tau_{iono}$ and $\tau_{trop}$ are the ionospheric and tropospheric errors, respectively. $\tau_{mul,\rho }$ is the multipath error of the code measurement, and $\varepsilon_{i,\rho }$ is the sum of the code measurement noise and other unmodeled errors.

Regarding the carrier-phase measurement, which is indeed the difference between the carrier phase of the incoming signal from the CAPS satellite and the carrier signal phase replicated by the user receiver, its observation model is shown in Equation (5), where the ambiguity is introduced to the observation:

(5) $$\lambda \cdot \phi^{{}_{i}}=r^{{}_{i}}+ct_{u}-ct^{{}_{i}}+\tau_{iono}-\tau_{trop}-\lambda \cdot N^{i}-\tau_{mul,\phi }+\varepsilon_{i,\phi }$$

where $\phi^{{}_{i}}$ is the carrier-phase measurement in cycles at the C1 frequency; λ is the wavelength of the CAPS navigation signal and $N^{i}$ means the ambiguity of the carrier-phase; $\tau_{mul,\phi }$ is the multipath error of the carrier-phase measurement; and $\varepsilon_{i,\phi }$ is the sum of the carrier-phase measurement noise and other unmodeled errors. This is similar to traditional GNSSs insofar as the carrier-phase observation can be used as a high-precision pseudo-range measurement when the ambiguity is correctly fixed.

To provide a more intuitive understanding of the sources of error in CAPS, a brief discussion of the errors in Equations (4) and (5) was given as follows:

$t_{u}$—is related to the receiver clock, and can be absorbed by the parameter of the clock bias during positioning.

$t^{{}_{i}}$—contains two parts: the model error of *T* which is usually smaller than 1 ns [10] and the ephemeris error which is about 2–3 m [3]. This ephemeris error reflected in the measurement is the projection on the vector from the satellite to the user.

$\tau_{iono}$—is the difference between the ionospheric delay from the satellite to the master control station and the ionospheric delay from the satellite to the user receiver. In addition, for the same propagation path, the ionospheric delay of C1 frequency is about 1/6 of the ionospheric delay of L1 frequency (inversely proportional to the square of the frequency [13]). Therefore, the ionospheric delay error of CAPS is much smaller than that of traditional GNSSs.

$\tau_{trop}$—is the difference between the tropospheric delay from the satellite to the master control station and the tropospheric delay from the satellite to the user receiver. The value is related to the location of the user receiver and to the atmospheric conditions.

$\tau_{mul,\rho }$, $\tau_{mul,\phi }$—are related to the surroundings of the receiver antenna. For the carrier-phase measurement, the effect of the multipath is much smaller for the C-band than that of the L band signal.

The doppler measurement is the difference of the observed frequency of a signal from the CAPS satellite and its corresponding nominal frequency, and this difference is generally the doppler shift caused by the relative motion of the receiver and satellite. The observation equation can be expressed as Equation (6):

(6) $$f_{d}^{i}=D\cdot (v^{{}_{i}}-v_{u})+cb+n_{d}$$

where $f_{d}^{i}$ is the doppler observation; D is the direction vector; $v^{{}_{i}}$ is the velocity of the ith satellite; $v_{u}$ is the velocity of the user; b is the frequency shift of the receiver clock; and $n_{d}$ is the noise of the doppler measurement. This is a linear equation and the velocity of the user can be directly calculated by the least square when tracking more than four satellites with a good Dilution of Precision (DOP) value.

### 2.3. Relative Positioning Model

The accuracy of the code and carrier-phase measurements are significantly affected by the errors from the orbit, virtual clock, ionospheric delay and tropospheric delay. Those errors can generally be eliminated or mitigated as much as possible by the differential observation between two neighboring receivers. Therefore, the differential observation model of the code and carrier-phase measurements between the user and base receivers can be expressed as:

(7) $$\left\{ \begin{array}{l} \Delta \rho^{{}_{i}}=\Delta r^{{}_{i}}+c(t_{u}-t_{b})+\Delta \tau_{mul,\rho_{i}}+\varepsilon_{\Delta \rho_{{}^{i}}}\\ \Delta \phi^{{}_{i}}=\Delta r^{{}_{i}}+c(t_{u}-t_{b})-\lambda \cdot \Delta N^{{}_{i}}+\Delta \tau_{mul,\phi_{i}}+\varepsilon_{\Delta \phi_{i}} \end{array}\right.$$

where Δ denotes the differential operation; and $t_{b}$ is the sum of the hardware delay and the clock error at the base receiver. $\Delta \tau_{mul,\rho_{i}}$ and $\Delta \tau_{mul,\phi_{i}}$ are the differential multipath errors for the code and carrier-phase measurements, respectively.

The errors from the orbit and virtual clock can be completely eliminated via the differential operation, while the ionospheric and tropospheric errors can only be mitigated as much as possible. The remaining ionospheric and tropospheric delay generally increase when the distance between two receivers increases. As discussed in Section 2.2, a smaller differential ionospheric delay error is very advantageous for extending the length of the baseline between the rover and base station.

Moreover, the carrier-phase observation can be considered as the high precise range measurement when the integer ambiguity $\Delta N^{{}_{i}}$ can be reliably resolved. The number of CAPS satellites was no more than 5 when we carried out our experiment, and the geometric distribution was not very good due to the 4 GEO satellites being located in east-west directions. Additionally, the receiver can only obtain the observation of a single frequency. These make it quite difficult to fix the ambiguities reliably and quickly based on the geometry-based algorithm in current GNSSs like LAMBDA [14] since this relies on the geometrical change caused by the satellites’ movement [15,16], while the GEO satellites are almost stationary relative to users in CAPS. In this study, the ambiguities were directly estimated using the geometry-free method via Equations (8) and (9).

(8) $$\Delta {\hat{N}}^{{}_{i}}=mean{\left(\frac{\Delta \rho^{{}_{i}}-\Delta \phi^{{}_{i}}}{\lambda }\right)}_{L}$$

(9) $$\Delta N^{{}_{i}}=[\Delta {\hat{N}}^{{}_{i}}]$$

where, $\Delta N^{{}_{i}}$ and $\Delta {\hat{N}}^{{}_{i}}$ are the fixed and float ambiguity, respectively; $\Delta \rho^{{}_{i}}$ and $\Delta \phi^{{}_{i}}$ means the series of differential code and carrier-phase observations during a continuous period; mean represents the average operation over a period of *L* epochs; and the square brackets [ ] represent rounding to the nearest integer. For Equations (8) and (9), the premise for obtaining the correct ambiguity is that the series $\Delta \rho^{{}_{i}}-\Delta \phi^{{}_{i}}$ is zero-mean. For the differential observations $\Delta \rho^{{}_{i}}$ and $\Delta \phi^{{}_{i}}$, the ionospheric delay, tropospheric delay, the model error of the virtual clock and the ephemeris error were almost completely eliminated under a zero-baseline or very short baseline. In this case, the main error sources which effect the fix of ambiguities are the multipath error and the observation noise of the code and carrier phase measurements. Assuming the observation noise of code and carrier-phase measurements obeys Gaussian distribution with zero-mean, the standard deviation of the float ambiguity can be described by:

(10) $$\sigma_{\Delta {\hat{N}}^{{}_{i}}}=\frac{1}{\sqrt{L}}\sqrt{\frac{\sigma_{\Delta \rho^{i}}^{2}+\sigma_{\Delta \phi^{i}}^{2}+\Delta \tau_{mul}^{2}}{\lambda^{2}}}$$

where $\sigma_{\Delta \rho^{i}}^{2}$ and $\sigma_{\Delta \phi^{i}}^{2}$ are the variance of the differential code and carrier-phase measurements, respectively; $\Delta \tau_{mul}^{}$ represents the total effect of the multipath on $\Delta \rho^{{}_{i}}-\Delta \phi^{{}_{i}}$; and L is the epoch number of averaging.

From Equation (10), it can be seen that, if the observation conditions are good enough that the code and carrier-phase measurements do not have multipath errors, the correct *N* can be obtained over a proper period and the epoch number L depends on the value of $\sigma_{\Delta \rho^{i}}^{2}$ and $\sigma_{\Delta \phi^{i}}^{2}$. In the presence of the multipath, the value of $\Delta N^{{}_{i}}$ will have an integer error, which depends on the size of the multipath. This error will eventually affect the positioning result; its impact on the positioning error will depend on the geometric factor corresponding to the satellite i. Therefore, and open observation environment was selected while installing the antenna in our experiment in order to minimize the impact of the multipath.

In addition, this equation is only a preliminary estimative approach for obtaining the integer value of the differenced ambiguity; however, further research is required on how to test and validate the ambiguity estimates with such a short wavelength.

## 3. Experiment Result and Analysis

The numerical experiment for validating the CAPS performance was carried out by analyzing the noise of the code and carrier-phase measurement based on zero-baseline data and by analyzing the accuracy of the relative positioning and velocity estimation in post-processing and real-time cases respectively based on short baseline data.

### 3.1. Analysis of the Measurement Noises

**Figure 5:**
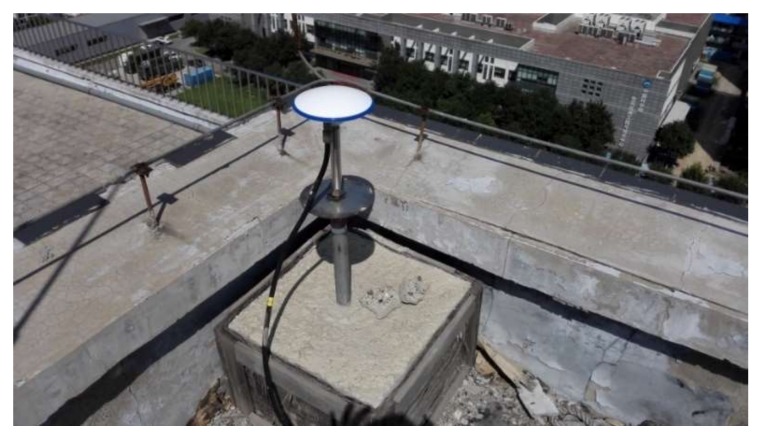
Surrounding environment of the antenna installed on the roof of the Academy of Opto-Electronics for the zero-baseline experiment.

The noise of the code and carrier-phase measurement can generally be assessed using the data from the static zero-baseline experiment with a common antenna [2,17]. The residual of the double-differenced observations on the inter-station and inter-satellite, in which the errors of the orbit, the bias resulting from the virtual clock offset model, ionospheric error, tropospheric error, receiver hardware delay and clock offset have been eliminated, is able to indicate the noise of the raw un-differenced code and carrier-phase measurement. We carried out the zero-baseline experiment tracking the CAPS navigation signal for more than four hours on 1 August 2017, with a 4-Hz sampling frequency, and the number of total epochs was over 70,000. Since CAPS are still in the development and testing phase, there is no commercial CAPS receiver yet. Two receivers developed by ourselves from scratch were used in this zero-baseline experiment. The basic structure of the self-developed receivers is almost like a GPS receiver. The largest difference is that the frequency of the coming signal is the C-band which means the carrier replicated by the CAPS receiver while acquisition and tracking is the C-band. There is no anti-multipath processing inside the current receiver. Those two receivers are connected to the same antenna via a power splitter. The antenna in our experiment was installed on the roof of the Academy of Opto-Electronics (AOE) in Beijing, China, and the CAPS navigation signals from the 4 GEO satellites and 1 IGSO satellite were tracked by our receivers during the experiment period. Since the observation environment is very open (see Figure 5), the multipath error can be considered at a relatively low level. For a receiver located in Beijing, the 4 GEO satellites are always visible while the IGSO satellite is visible for about 10 h every day. During the experiment, the visible time in Beijing is about 6:00 to 16:00 local time, and it cannot be seen during the rest of the time, which makes the three-dimensional solution impossible. The data in the experiments was collected from 9:00 to 14:00. During this time, the elevation angle of the IGSO was relatively high, which made the geometric configuration relatively good.

**Figure 6:**
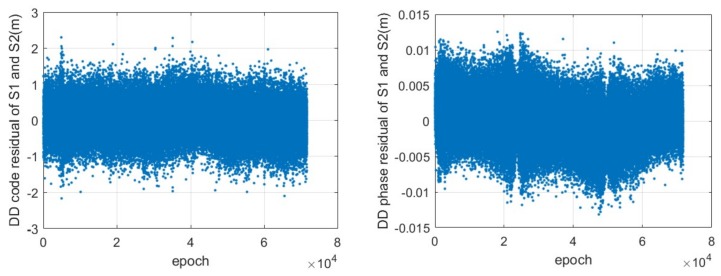
Time series of the residuals of the double-differenced code (**left**) and carrier-phase (**right**) measurements between the CAPS satellites S1 and S2 with a sampling rate of 4 Hz.

As an example, the series of double-differenced residuals between the two CAPS satellites S1 and S2 are shown in Figure 6. The standard deviation (STD) of the residual from all pairs of CAPS satellites are illustrated in Table 1. The STD of the residuals of the double-differenced code measurements ranges from 0.42 to 0.57 m, while the STD of the residuals of the double-differenced carrier-phase measurements varies between 0.13 and 0.34 cm. Providing that the STDs of the un-differenced measurements are identical for different CAPS satellites and that they are independent from each other, we can infer that the noise of the code measurements is about 0.25 m, and that the noise of the carrier-phase measurement ranges from 0.07 to 0.17 cm. The high accuracy of the carrier-phase measurement is advantageous for further improving the CAPS precise positioning.

### 3.2. Post-Processing Experiment

In this post-processing experiment, the relative positioning and velocity estimation was carried out based on a very short baseline in static mode from 9:00 to 14:00 local time, on 1 August 2017, which is the same time period as for the zero-baseline described in Section 3.1.; there are more than 70,000 epochs in the data collection. Two antennas were installed on the roof of AOE, one of which is the same antenna mentioned in Section 3.1, while the other antenna was about 6 m away from the first one. These two right-hand circularly polarized microstrip antennas are specially designed for the evaluation and processing of the CAPS signal. They can receive the L-band and C-band signals simultaneously and have passed the microwave darkroom test. The standard antenna test results showed that the offset of the phase centers of L-band and C-band can be almost considered coincide in the horizontal component and it is about several millimeters in the vertical component. In our experiment, this mm-level offset of phase center between the signals of C- and L-band has been ignored in comparison with the cm-level positioning validation. The position of the antenna used in our experiment was precisely estimated over a long enough period of averaging of the estimated position series using the RTK technology based on GPS with a nearby reference station in the reference frame of ITRF2000, which is in line with CAPS03; it could therefore be considered the reference standard. The real velocity could be considered to be zero.

The ground track of the CAPS satellites at the time of our experiment is shown in Figure 2. The code and carrier-phase measurements were individually used in the relative positioning, and the doppler measurement was used for the velocity estimation. The carrier-phase ambiguities in the relative positioning were fixed by the approach described in Section 2.

Using the results in Section 3.1, we can analyze the ambiguity estimation error in Equation (9) and determine the value of the smooth period L. Since the trend term error caused by the multipath cannot be eliminated by smoothing, we first determine the value of $\sigma_{\Delta N^{{}_{i}}}$ in the absence of the multipath error. Assuming that the residual of the float ambiguity obeys the zero-mean Gaussian distribution and that the observations in two neighboring epochs are independent from each other, if $3\sigma_{\Delta N^{{}_{i}}}<0.5$, the correct ambiguity can be obtained with a probability of 99.73% through the rounding algorithm. For the sake of conservation, we take the standard deviations of the code and carrier phase measurement noise as 0.25 m and 0.17 cm, respectively. According to Equation (10), a period averaging approximately 3 min is needed while the sampling rate is 4 Hz. If one takes the multipath error into account, the mean of the residual may be non-zero. Taking the multipath error of 1 m as an example, the mean of the ambiguity residual distribution will deviate by a 0.5 cycle, which will cause a 1 cycle error on $\Delta N^{{}_{i}}$ with a 50% probability. The integer error on $\Delta N^{{}_{i}}$ will affect the positioning result, and its impact on the positioning error will depend on the geometric factor corresponding to the satellite i. In our experiment, six minutes of data were used; the data series from the first three minutes were used to fix an initial ambiguity, while the ambiguity fixed with the last three minutes was used for verification. If the value of these two ambiguities was the same, then the integer value was considered to be the correct ambiguity.

**Figure 7:**
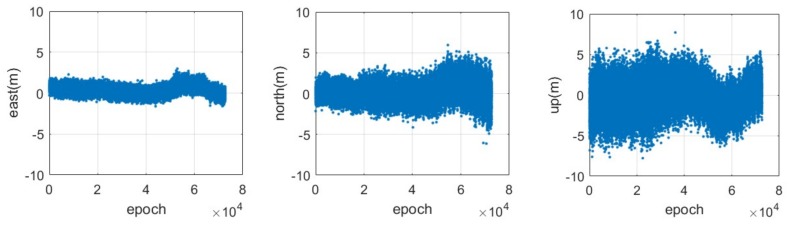
Time series of the positioning error in the east (**left**), north (**middle**) and up (**right**) components for the CAPS static relative positioning based on the code measurement with a sampling rate of 4 Hz.

**Figure 8:**
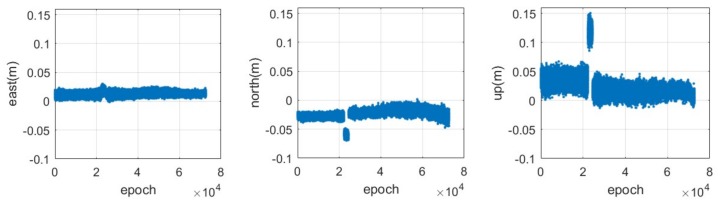
Time series of the positioning error in the east (**left**), north(**middle**) and up (**right**) components for the CAPS static relative positioning based on the carrier-phase measurement with a sampling rate of 4 Hz.

The positioning error can be calculated from the differences of the rover coordinates between the value estimated from the CAPS relative positioning and the ‘real’ value. The distribution of the series of positioning errors in the east, north and up components based on the code measurements are illustrated in Figure 7, while the corresponding results for the carrier-phase measurements are provided in Figure 8, respectively. It can be seen that the positioning error in the east-west component is much smaller than that in the south-north and up components, both for the code and for the carrier-phase measurements. This is mainly because of the distribution of the current CAPS constellation, including the 4 GEO satellites and 1 IGSO satellite in view, as the 4 GEO satellites are located in the east-west direction (See Figure 2). It can be seen from Figure 7 that there is an obvious slow fluctuation in the differential positioning result based on the code measurement. This is caused by the multipath error. Although we chose, to the extent that was possible, an open area when placing the antenna, the effects of the multipath still could not be avoided completely. This error would be reduced with the addition of anti-multipath processing in our CAPS receiver in future research.

Moreover, it can be seen from Figure 8 that the distribution of the positioning error is not zero-mean and that it may have occasional jumps. The non-zero-mean of the positioning error is due to the integer bias on the ambiguities which is caused by the multipath error. The reason for those jumps may could line in a twice cycle slip that may have occurred but failed to be detected during the period. This result infers that we must take care of the ambiguity of the CAPS carrier-phase due to the short wavelength compared with that of GPS or BDS.

The root mean square (RMS) of those positioning errors is calculated using the result during the experiment period, shown in Table 2. The accuracies of the CAPS relative positioning based on the code measurement are about 0.66, 1.07 and 1.8 m in the east-west, north-south and vertical components, while they are about 1.35, 2.49 and 3.15 cm for the result based on the carrier-phase. Following our experience, the precision of the differential CAPS using the code and carrier-phase measurements is almost at the same level as current BDS and GPS [18]. In addition, we are able to calculate the STDs of the relative positioning error based on the carrier-phase measurement in three different components by removing the averaged value of the positioning errors, and they are about 0.33, 0.46 and 0.81 cm for the three components, respectively. This may likely be regarded as the precision of the relative positioning with the carrier phase when the ambiguities are correctly fixed.

**Figure 9:**
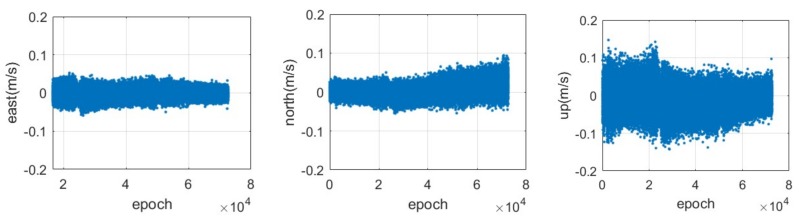
Time series of the error in the east (**left**), north (**middle**) and up (**right**) components for the CAPS velocity estimation based on the doppler measurement with a sampling rate of 4 Hz.

The time series of the velocity estimation error in the three components are shown in Figure 9, where the true value is considered to be zero. Similar to the result of the positioning error, it shows a high accuracy in the east-west component due to the distribution of the current CAPS constellation, followed by the north-south and vertical components. The RMS of the velocity estimation is illustrated in Table 2, and we are able to determine that the level of accuracy can reach about 1.35, 1.70 and 3.23 cm/s in the east-west, north-south and vertical components, respectively.

### 3.3. Real-Time Experiment

In addition to the post-processing experiment, we also carried out a real-time kinematic experiment based on the carrier-phase in order to validate the performance of the CAPS in cm-level positioning. The reference antenna was installed on the roof of AOE at the same location as that shown in Figure 5, and its position was precisely determined by GPS using the RTK approach described in Section 3.2. Based on the raw measurements from the reference receiver, the corrections of the code and carrier-phase and their variation rate could be calculated using the known position of the reference antenna, before being broadcast to the user via radio. The rover received these corrections and used them to correct the error of observation during the positioning estimation. The maximum distance between the reference station and rover was around 8.2 km.

During our test, the sampling rate of the reference receiver and rover receiver was 4 Hz, and the differential correction from the reference station was broadcast at 1 Hz. The experiment was carried out on the morning of 24 August 2017, and the 4 GEO and 1 IGSO satellites could be tracked by our self-developed receiver in Beijing, China. Moreover, the velocity was also measured during the real-time experiment. The experiment was divided into two parts: first, the test vehicle was parked off the road for about 15 min after the initial ambiguities were fixed and began to move on the road for about 25 min with a speed of 40–60 km/h. The NovAtel GPS/BDS RTK and INS integrated terminal was installed with a common antenna with the user receiver in order to obtain the ‘true’ coordinates and velocity to validate the accuracy of the relative positioning. The nominal accuracy of the ‘true’ coordinates and velocity generated by the NovAtel terminal are 1–2 cm and 0.02 m/s, respectively. It should be noted that the true velocity for the rover during the static period was selected to be zero for the validation rather than the result from the NovAtel terminal.

**Figure 10:**
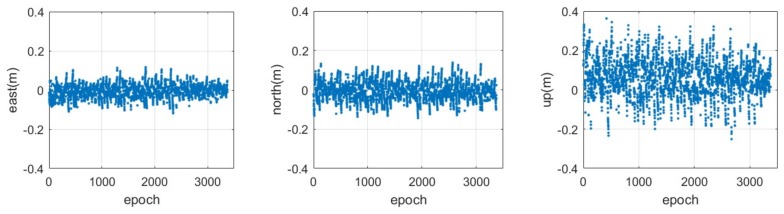
Time series of the positioning error in the east (**left**), north (**middle**) and up (**right**) components for the real-time CAPS relative positioning based on the carrier-phase in static mode with a sampling rate of 4 Hz.

**Figure 11:**
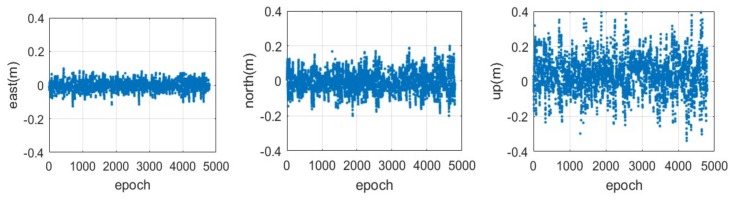
Time series of the positioning error in the east (**left**), north (**middle**) and up (**right**) components for the real-time CAPS relative positioning based on the carrier-phase in kinematic mode with a sampling rate of 4 Hz.

The time series of the positioning error in the three components during the static period is illustrated in Figure 10, and the corresponding result during the kinematic period is shown in Figure 11. Compared with the post-processing result, we can find that the CAPS static relative positioning based on the carrier-phase becomes a little poor in these three components. Moreover, the real-time kinematic positioning performance of CAPS in the horizontal and vertical components is much poorer. The RMS of the static differential CAPS positioning based on the carrier-phase is about 2.83, 4.37 and 10.41 cm in the east-west, north-south and vertical components, while they are about 3.33, 5.36 and 10.78 cm for the kinematic mode.

**Figure 12:**
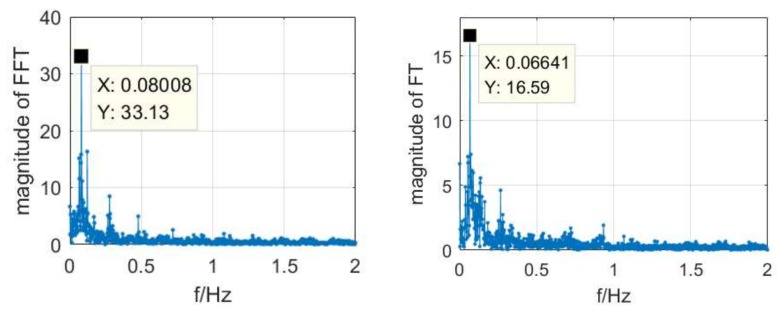
The spectral analysis result of the east positioning error in static (**left**) and in dynamic (**right**) modes with FFT.

It can be seen from Figure 10 and Figure 11 that the position result shows some periodic feature, while the post-processing result doesn’t. A spectral analysis of the east positioning error in static and dynamic modes with FFT was shown in Figure 12. The largest magnitude was located at 0.08 Hz in static mode, which means a period of about 12.5 s. A similar result was obtained from the positioning errors of other components. In dynamic mode, the largest magnitude was located at 0.066 Hz, which means a period of about 15 s. The reason for this may lie in the error model of the corrections, in the truncation error or in the errors related to the time delay of the broadcast. The specific reasons still need further analysis and more experiments. We can infer that the positioning accuracy would be improved once this problem was solved.

**Figure 13:**
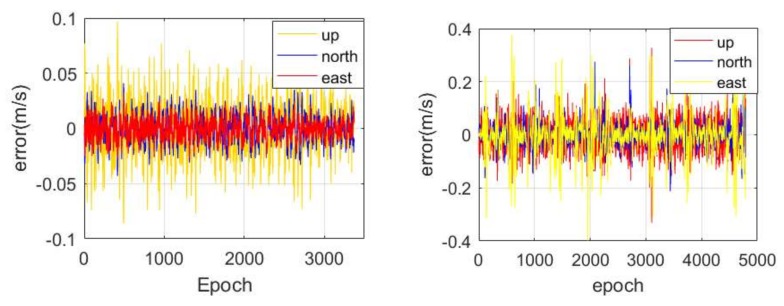
The error of the velocity estimation based on CAPS in static (**left**) and kinematic (**right**) modes with a sampling rate of 4 Hz.

The error of the CAPS velocity estimation in the static and kinematic modes are given in Figure 13. We find a similar phenomenon with the positioning error, i.e., that the error in the east-west component is much smaller than that in the other two components. This phenomenon mainly resulted from the current distribution of the CAPS constellation. The error RMS of the velocity estimation in the east-west, north-south and vertical components are about 0.01, 0.013 and 0.026 m/s during the static period, while they are about 0.08, 0.051 and 0.051 m/s during the kinematic period, respectively. In addition, it should be noted that the error of the reference velocity based on the NovAtel terminal has also been absorbed in the RMS calculation, which may make the accuracy of the CAPS velocity estimation in kinematic mode looks slightly worse.

## 4. Conclusions

CAPS is the new navigation concept proposed by the Chinese Academy of Science for establishing a navigation system based on communication satellites. It is currently regarded as an experimental verification platform supporting the design, construction and update of BDS. Based on the real data collected in Beijing, this contribution analyzed the noise of code and carrier-phase measurements and showed the first results of relative CAPS positioning and velocity estimation in post-processing and real-time experiments. The following conclusions can be draw from our numerical experiment.

(1) The noise of the code and carrier-phase measurement from CAPS is about 0.25 m and 0.13 cm, respectively.
(2) The accuracies of static relative CAPS positioning based on the code measurement is about 1.26 and 1.80 m in the horizontal and vertical components, while they can be improved to 2.83 and 3.15 cm when using the carrier phase via the ambiguity fixed by the geometry-free approach. The velocity estimation with doppler measurements of CAPS has an accuracy of about 0.04 m/s in the static experiment.
(3) The accuracy of kinematic relative CAPS positioning based on the carrier-phase is about 5.21 and 10.41 cm in the horizontal and vertical components, and the accuracy of the velocity estimation is about 0.11 m/s.
(4) The relative positioning results in the real time experiment show a periodic feature which needs further analysis with more experiments.

Since CAPS is currently still in the development and test phase, this paper only provides preliminary results on relative CAPS positioning and velocity estimation based on limited data and experiments. It should be noted that the data and experiments in this work are quite insufficient to fully represent the performance of CAPS. This is because some of the satellites used in CAPS were rented on a short-term basis, and additionally the ground control station is still being tested. Moreover, the continuous and stabilized service is not available yet, so the available data is limited. With the further improvement of the CAPS system, the collection of longer data and various experiments will expand throughout China in the near future. In addition, we will also compare the positioning result between CAPS and current GNSSs like BDS, GPS, etc.

## Figures and Tables

**Table 1:** STD of the residuals of the double differenced code and carrier-phase measurements between the two CAPS satellites.

	Satellite Pairs	S1–S2	S3–S2	S4–S2	S5–S2
Measurement					
Code (m)		0.56	0.49	0.48	0.42
Carrier-phase (cm)		0.34	0.24	0.13	0.22

**Table 2:** Root Mean Square (RMS) of the CAPS relative positioning and the velocity estimation during the experiment period for three components.

	Components	East-West	North-South	Vertical
RMS				
Relative positioning	Code (m)	0.66	1.07	1.80
	Carrier-phase (cm)	1.35	2.49	3.15
Velocity estimation (cm/s)		1.35	1.70	3.23
